# The Effects of a Short Foot Exercise Device on Foot Posture, Pain, Balance, and Walking Speed for Individuals With Foot Pain

**DOI:** 10.1002/jfa2.70238

**Published:** 2026-11-06

**Authors:** Yale B. Hughes, Ashlyn M. Jendro, Avery G. Dilley, Elizabeth A. Parke, Abigail C. Schmitt

**Affiliations:** ^1^ The MOVE Lab, Exercise Science Research Center, Department of Health, Human Performance, and Recreation University of Arkansas Fayetteville Arkansas USA

**Keywords:** biomechanics, center of pressure, gait, intrinsic foot muscles, pes planus

## Abstract

**Introduction:**

Weak intrinsic foot muscles may lead to foot pain, altered foot posture, single‐leg balance issues, and altered gait. Short foot exercises can strengthen intrinsic foot muscles, which can improve foot posture and single‐leg balance. This study tested the efficacy of a training device designed to strengthen intrinsic foot muscles, the FootCore MAX, for decreasing pain, improving single‐leg balance, improving foot posture, and increasing comfortable walking speed.

**Methods:**

Thirteen participants with chronic foot pain (10 female; 25.4 ± 4.3 yrs; body mass index 26.7 ± 6.8) performed 6 weeks of at‐home short foot exercise using the FootCore MAX training device. Pre‐ and post‐training visits included questionnaires, single‐leg balance testing, foot posture measures, and comfortable and fast walking speeds. Participants also rated their foot pain before and after each lab visit.

**Results:**

Foot and Ankle Outcome Score pain subscore decreased 30% (37 ± 18 to 26 ± 15, *p* = 0.040) after 6 weeks of training. Foot and Ankle Disability Index scores decreased 25% (4 ± 4 to 4 ± 3, *p* = 0.032), indicating disability decreased. Foot and Ankle Ability Measure total scores improved by 14% (72 ± 15 to 82 ± 16 *p* = 0.015), indicating ability improvement. Visual analog pain rating scores showed a significant interaction between both the beginning and end of lab visits as well as pre‐ and post‐training (*p* = 0.002), suggesting pain scores were influenced by the training, as pain scores significantly increased from the beginning to the end of the pre‐training lab visit (12 ± 11 to 27 ± 14, *p* = 0.001), but not from beginning to end of the post‐training lab visit (14 ± 13 to 16 ± 18, *p* = 0.314). Preferred gait speed significantly increased with training (1.06 ± 0.33 to 1.16 ± 0.20, *p* = 0.008). Neither fast walking speed, balance, or foot postural measurements changed from pre‐ to post‐training (*p* > 0.05).

**Conclusion:**

Six weeks of short foot exercise training with the FootCore MAX can improve foot pain and increase comfortable gait speed.

## 1 Introduction

Twenty to 37 percent of the population have pes planus, or flat feet, the clinical combination of collapsed medial longitudinal arch (MLA), rearfoot eversion, and forefoot abduction [1, 2]. Pes planus can be painful and is associated with lower limb pathologies, including patella‐femoral pain, plantar fasciitis, chronic ankle instability, tibialis posterior disfunction, and hallux valgus [1, 3, 4, 5, 6, 7, 8, 9]. Foot pain can diminish quality of life, reduce physical activity, decrease gait velocity, and decrease independence [10, 11, 12]. Orthotic inserts are often prescribed by clinicians for the treatment of pes planus or foot pain and, while orthotics can improve pain, research indicates that even short‐term use of orthotics can cause atrophy of the intrinsic foot muscles (IFM) [13].

IFM, the small muscles of the foot, help maintain the structure of the MLA [6, 14, 15]. During the weight acceptance and push‐off portions of the stance phase in the gait cycle, the MLA of the foot acts as a cushion during impact and a spring during push‐off [6, 15]. In addition to acting as a cushion and spring, IFM assist in maintaining balance, especially during single‐leg balancing. When postural demands increase from double‐leg to single‐leg stance, recruitment of IFM also increases [16], acting to stiffen the arch of the foot to assist in maintaining static balance [17]. Thus, altered IFM activity could impact an individuals' stability [16, 17, 18].

One way to strengthen the IFM is short foot exercises (SFE). SFE involves shortening the foot and raising the arch by pulling the heads of the metatarsals toward the heel, without curling the toes [18]. When these muscles contract, shortening the foot, the height of the MLA increases [15]. SFE independently recruits IFM without recruiting extrinsic foot muscles and has been shown to decrease pain in populations with plantar fasciitis, chronic ankle instability, pes planus, and patellofemoral pain [9, 14, 18, 19, 20]. SFE can also improve balance, improve foot posture, and decrease foot pain [1, 9, 14, 18, 21, 22].

While SFE improve balance, foot posture, and pain, little is known about the effects of SFE on gait spatiotemporal measurements. Gait velocity is known to decrease with foot pain in older populations [12] as well as in young adult populations [11]. However, to our knowledge, the relationship between an SFE training program and walking gait velocity and foot posture has only been investigated with distance runners [23]. An overarching issue in SFE research is the lack of protocol standardization for training methodology; varied interventions and approaches to SFE create a need for a standardization [1]. Furthermore, despite widespread use in foot therapy, SFE is considered to be a more difficult motor skill to learn because it requires selective activation of the IFM without flexion of the most distal metatarsal joints [6]. Previous studies have investigated electromyographical and ultrasound feedback methods to facilitate learning SFE; however, these biofeedback methods are complicated and expensive, rendering them ineffective for daily clinical practice [24, 25]. A relatively inexpensive and easy‐to‐use device which standardizes the approach to SFE and gives immediate feedback to participants could help fix this issue. The FootCore MAX (BodyCore Technologies, Bentonville, Arkansas) is a device that replicates SFE and is advertised to strengthen the IFM with only 4 minutes of use—two minutes each foot—per day. This is important for standardization of SFE but has deeper implications for populations living with foot pain. If the FootCore MAX works as advertised, individuals living with foot pain could confidently use this device at home or work to strengthen their IFM, improve their MLA function, and decrease pain. However, the efficacy of the FootCore MAX for improving foot pain, improving pes planus related foot posture measures, improving (i.e., increasing) gait speed, and improving single‐leg balance is unknown.

Thus, the purpose of this study was to determine the effects of training with the FootCore MAX on foot pain, foot posture measures associated with pes planus—navicular drop (ND) and arch height index (AHI), gait velocity, and single‐leg balance in people with self‐reported pes planus or foot pain. We hypothesize that 6 weeks of training with the FootCore Max device will increase walking speed and improve foot posture by decreasing ND, increasing AHI, seated and standing, and decreasing the rearfoot eversion angle. We also hypothesize that self‐reported pain will decrease, single‐leg static balance will improve, and comfortable and fast walking speeds will increase after the training program.

## 2 Methods

Participants were recruited through fliers and email announcements and were screened for eligibility. Inclusion criteria were as follows: ages between 18 and 30 years and self‐reported foot pain, flat‐feet or pes planus, plantar fasciitis, or general foot dysfunction. Individuals were excluded if they presented with a lower limb length deformity, physical foot deformity (e.g., hallux rigidus, hammer toes), or neuropathic ulceration. Individuals were also excluded if they had a history of a diagnosed neurological or orthopedic condition or musculoskeletal injury that impeded their ability to safely participate in the walking tasks. Individuals were excluded if they had loss of vision, presence of an unstable medical, or psychiatric condition that may preclude their ability to safely participate or follow directions. Exclusion criteria also included the presence of untreated cardiovascular disease, pulmonary disease, or any disease or recent health change that disrupts balance or motor control. If participants were still eligible after screening, they were asked to come to the MOVE Laboratory (University of Arkansas) for further evaluation. All participants provided electronic consent prior to any study procedures, and the study was approved by the University's Institutional Review Board (protocol #: 2108353831). Out of 62 people who completed the eligibility survey, 15 participants agreed to participate in the study. Two participants were excluded from data analysis due to withdrawing from the study prior to the final data collection. Thus, 13 participants with self‐reported foot pain performed 6 weeks of at‐home SFE using the FootCore MAX training device.

During laboratory visits, participants were evaluated for (1) foot pain and function, (2) foot posture and function, (3) walking speed, and (4) single‐leg balance. Questionnaires for foot pain and function included the Foot and Ankle Outcome Score (FAOS), the Foot and Ankle Disability Index (FADI), Foot and Ankle Ability Measure (FAAM) and a visual analog pain scale (VAS) [26, 27]. Participants reported their current pain level using a VAS at the beginning and end of each lab visit, for both pre‐ and post‐training sessions. Foot posture and function were assessed barefoot via ND and AHI standing and AHI seated. ND was measured as the difference in navicular tuberosity position between nonweight bearing (i.e., seated) versus weight bearing (i.e., standing) positions [28]. AHI was calculated as a ratio of dorsum height to truncated foot length, reported as a percentage [29]. Dorsum height is the vertical height of the foot measured in seated and standing positions (seated for AHI‐seated, standing for AHI‐standing) using a modified carpenter's square. Truncated foot length is the distance from the most posterior point of the calcaneus to the center of the first metatarsal head [29]. All foot postural measurements were obtained from a single trained evaluator to ensure consistency and reduce inter‐rater variability.

Following measurements, single‐leg static balance was assessed twice for each limb, for 30 seconds each [30, 31], on an embedded force platform (1000 Hz; AMTI Optima 600600, AMTI, Watertown, MA, USA). Participants were fitted with 42 reflective markers, by a single researcher, using the Oxford Foot Model and Lower Body Plug‐in Gait marker set to track 3D movement using a 16‐camera Vicon motion capture system (100 Hz; Vicon Motion Systems, Oxford, UK). Following a static calibration trial, participants were asked to complete 20 unobstructed walking trials on an 8‐m overground walkway while barefoot. The first 10 trials were completed at the participant's preferred, comfortable pace followed by another 10 trials at their fastest, yet safe, walking speed. Ten trials were recorded for each condition to maximize the reliability of kinematic data [32].

Following the balance and walking tasks, markers were removed, and participants were given a FootCore MAX device (Figure 1). A single researcher fitted the devices to each participant by adjusting the position of the sliding paddles and taught participants how to use the FootCore MAX device by placing their heel at the back of the device, in the heel cup, and placing the distal heads of the metatarsals on the sliding paddle. The researcher then demonstrated the movement by asking participants to shorten their foot by trying to pull the heads of the metatarsals toward their heel without allowing their toes to curl or leave the device. Following a successful demonstration, participants were asked to use the device daily for 2 minutes per foot, at a self‐selected repetition pace, for a total of 6 weeks. No additional instructions about the training were provided. Participants returned to the lab halfway through the training for a check‐in visit to ensure proper technique. Training program adherence was self‐reported categorically as *daily*, *frequently*, *sometimes*, *rarely*, or *never*.

**FIGURE 1:**
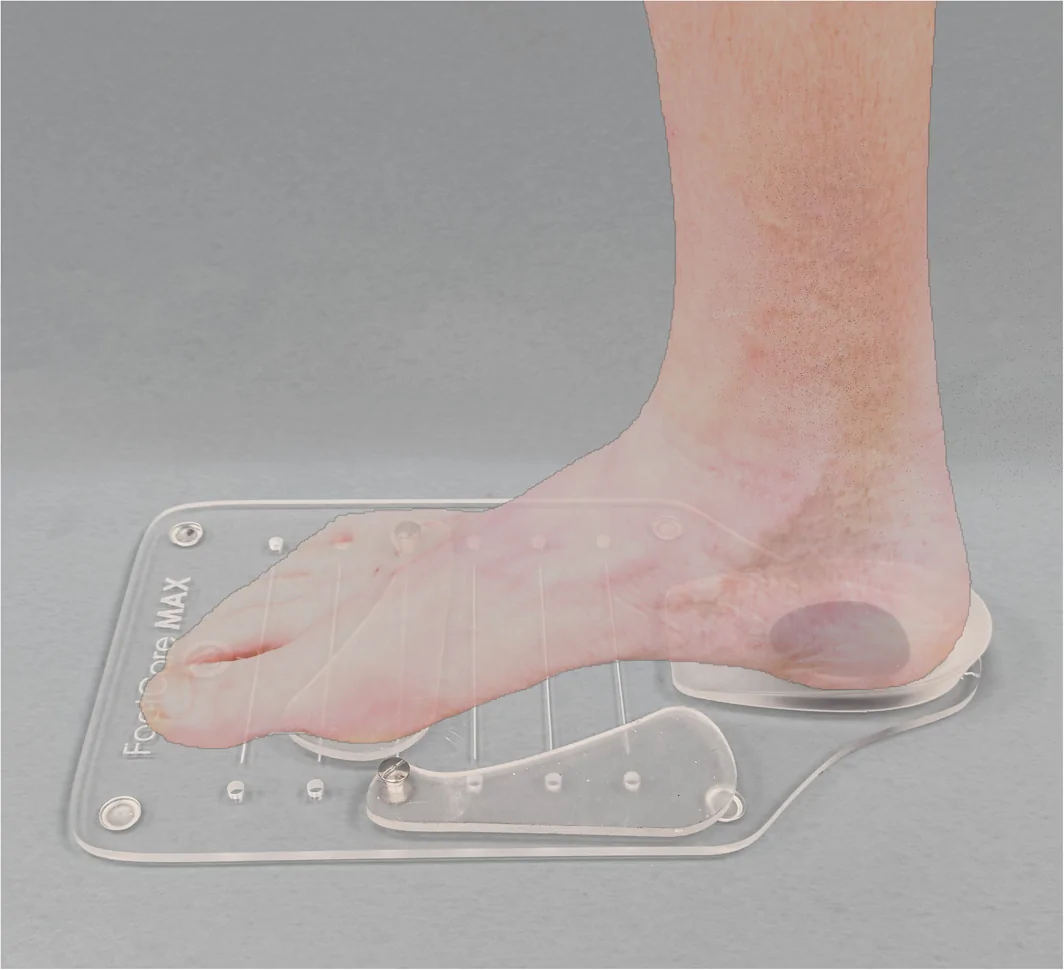
The FootCore MAX Training Device. The heel is positioned on the heel cup and the distal heads of the metatarsals rest on the sliding paddles while participants slide the paddle toward their heel by shortening their foot.

Following data collection, marker trajectory data were gap‐filled and filtered using a fourth order zero‐lag lowpass Butterworth filter at 6 Hz prior to model calculations. Data were exported and variables of interest were calculated and extracted using a custom MATLAB code (Mathworks, Natick, MA, USA, v2021b). The rearfoot eversion angle was determined as the average relative angle of the hindfoot relative to the tibia during the static trial where participants stood barefoot in a comfortable stance for one second. Gait speeds were calculated in MATLAB using the central difference method for the mean velocity of the center of mass averaged for all trials in each walking condition. Only participants who were able to balance for 30 seconds on each limb were included in the analysis of center of pressure (COP) single‐leg balance data. COP data were down sampled to 100 Hz for computing root mean squared distance in the anteroposterior (AP) and mediolateral (ML) directions, COP mean velocity in the AP and ML directions, and overall sway area [33]. COP data were averaged across trials and limbs for final data analysis.

Normality of the pre‐ to post‐test differences was assessed with Shapiro–Wilk tests and visual inspection of Q‐Q plots. There were no significant nonnormality nor were there extreme outliers among any variables, therefore a series of paired samples t‐tests was used to compare pre‐ and post‐training: (1) FAOS pain subscore, (2) FADI pain subscore, (3) FAAM, (4) ND, (5) AHI standing, (6) AHI seated, (7) rearfoot eversion angle, (8) preferred gait speed, (9) fast gait speed, and (10–15) COP‐based balance measures (RStudio v 4.4.2). Repeated measures analysis of variance (RM‐ANOVA) assessed within subject differences in VAS self‐reported pain levels for the beginning and end of lab visits at both pre‐ and post‐training sessions. *Post hoc* pairwise t‐tests with Bonferroni corrections, compared all four VAS conditions (session: beginning of lab visit and end of lab visit, by training: pre‐training and post‐training). The level of significance for all statistical analysis was set at *p* < 0.05.

## 3 Results

Thirteen individuals with chronic foot pain completed the study (10 female; 25.4 ± 4.3 yrs; 1.65 ± 0.08 m; 73 ± 17.6 kg; body mass index 26.7 ± 6.8). Participant adherence to the FootCore MAX protocol was self‐reported by 11 out of 13 participants; adherence data were unavailable for 2 participants. Three participants self‐reported using the device *sometimes*, 5 participants reported *frequently*, and 3 participants reported *daily* use. No participants reported device usage as *never* or *rarely*.

Pain subscores from the FAOS and FADI significantly decreased from pre‐to post‐training; participants reported significantly less foot pain post‐training (Table 1). The FAAM total score and the FAAM function rating also significantly improved (higher scores represent greater foot function) from pre‐to post‐training (Table 1). The VAS showed a significant interaction between both beginning and end of lab visits as well as pre‐ and post‐training (*p* = 0.002, Table 2), suggesting changes in VAS pain scores were influenced by the training (Figure 2). A significant main effect of session (*p* = 0.012, Table 2) indicated that pain ratings increased from the beginning to the end of lab visits. *Post hoc* comparisons revealed that VAS scores significantly increased from the beginning of visit (*M* = 12 ± 11) to the end of visit (*M* = 27 ± 14) during the initial (pre‐training) session (*p* = 0.001). In contrast, there was no significant increase in pain from beginning of visit (*M* = 14 ± 13) to the end of visit (*M* = 16, ± 18) during the final (post‐training) session (*p* = 0.314). After participating in a 6‐week SFE training program with the FootCore MAX device, the pain increase associated with the barefoot tests in the post‐training lab visit was much smaller than pre‐training. This suggests that, while pain increased over the course of the pre‐intervention session, the intervention may have led to reduced movement‐evoked pain in the post‐intervention session (see Figure 2).

**TABLE 1:** Means and standard deviations of foot pain and function questionnaires (*n* = 13).

	FAOS pain subscore	FADI pain subscore	FAAM total score (%)	FAAM function rating
Pre‐training	37 ± 18	4 ± 4	72 ± 15	81 ± 14
Post‐training	26 ± 15	3 ± 3	82 ± 16	88 ± 11
*p*‐value	0.040*	0.032*	0.015*	0.014*

*Note:* Pre‐versus post‐training significance (*p* < 0.05) indicated with *.

Abbreviations: FADI, Foot and Ankle Disability Index; FAAM, Foot and Ankle Ability Measure; FAOS, Foot and Ankle Outcome Score.

**TABLE 2:** Repeated measures ANOVAs for VAS pain scores (*n* = 13).

Effect	df	F	*p*‐value	η^2^
Time × Session	1, 12	15.00	0.002*	0.05
Pre‐ and post‐training (training)	1, 12	0.96	0.346	0.02
Beginning and end of lab visit (session)	1, 12	8.65	0.012*	0.09

*Note:* Significant interaction (*p* < 0.05) indicated with *.

Abbreviations: df, degrees freedom; η^2^, eta squared.

**FIGURE 2:**
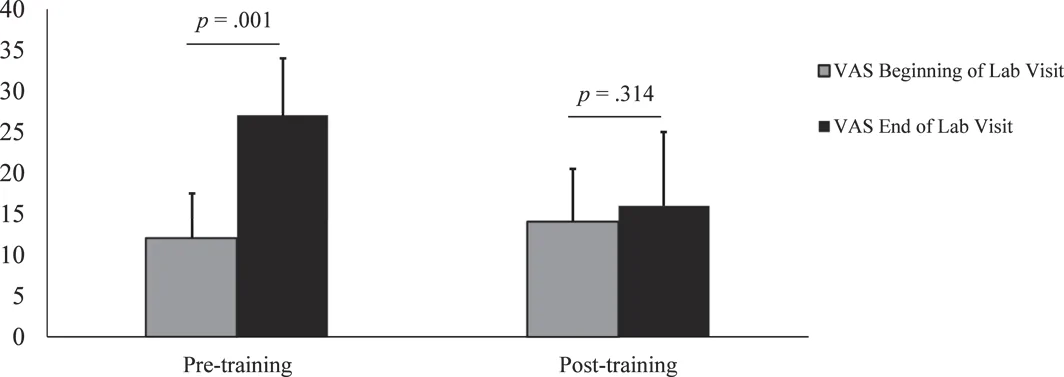
Pre‐ versus Post‐training VAS Pain Scores for Beginning and End of Laboratory Visits (*n* = 13). *Note.* Means and SD of VAS pain scores at the beginning and end of laboratory visits for both pre‐training and post‐training sessions. A significant interaction (*p* = 0.002) and main effect of session (*p* = 0.012) were observed using repeated‐measures ANOVA.

Six of the 13 participants were able to balance for the full 30 seconds on a single limb both pre‐ and post‐training, resulting in 7 participants being excluded for the final analysis of single‐leg balance data. Specifically, 7 participants successfully completed the single‐leg balance task pre‐training, and 7 participants successfully completed the balance task post‐training, with one participant gaining the ability to complete the task and one participant failing to complete the task post‐training. No significant differences were detected among balance measures when comparing pre‐to post‐training for COP‐based measures of single‐leg balance (*p* > 0.05, Table 3). Twelve out of 13 participants were used for statistical analysis for AHI because of an error in data collection. According to self‐reported data in the initial visit, 4 participants reported as having been diagnosed with flat feet or pes planus. However, during pre‐training measurements, 6 participants (46%) had at least one foot with greater than 10 mm of ND (the cut off for flexible flat foot) [34]. No significant differences were shown in ND, rearfoot eversion angle, AHI (for seated and standing), or fast gait speed when comparing pre‐to post‐training (*p* > 0.05, Table 4). In contrast, preferred gait speed significantly increased from pre‐ (*M =* 1.06 ± 0.33 m/s) to post‐training (*M =* 1.16 ± 0.20 m/s; *p* = 0.008).

**TABLE 3:** Means and standard deviations for single‐leg balance measures (*n* = 6).

	RMS AP (mm)	RMS ML (mm)	Velocity ML (mm/s)	Velocity AP (mm/s)	Sway area (mm^2^/s)
Pre‐training	7 ± 1	66 ± 1	23 ±	424 ± 5	6106 ± 30
Post‐training	88 ± 1	6 ± 0.3	424 ± 4	323 ± 1	10110 ± 14
*p*‐value	0.433	0.414	0.741	0.563	0.749

*Note:* Only 6 out of 13 participants successfully completed the single‐leg balance analysis. No significance was found pre‐ versus post‐training (*p* < 0.05).

Abbreviations: AP, anteroposterior; ML, mediolateral; mm, millimeters; RMS, root means squared; s = second.

**TABLE 4:** Foot postures and gait speeds (*n* = 13^a^).

	ND (mm)	RA (deg)	AHI‐seated (%)⁑	AHI‐standing (%)⁑	Preferred speed (m/s)	Fast speed (m/s)
Pre‐training	8 ± 5	0.62 ± 12.19	37 ± 3	34 ± 3	1.06 ± 0.33	1.61 ± 0.33
Post‐training	8 ± 5	5.85 ± 14.36	37 ± 4	34 ± 3	1.16 ± 0.20	1.62 ± 0.37
*p*‐value	0.816	0.080	0.969	0.834	0.008*	0.601

*Note:* Means and standard deviations of navicular drop (ND), rearfoot eversion angle (RA), arch height index (AHI). Pre‐ versus post‐training significance (*p* < 0.05) indicated with *.

^a^ For AHI‐seated and AHI‐standing, *n* = 12, due to data collection error for one participant.

## 4 Discussion

In this study, we hypothesized that 6 weeks of training with the FootCore MAX would improve self‐reported foot pain and in participants with pes planus or foot pain, as well as improve foot posture, improve single‐leg balance, and increase gait speed. Reductions in FAOS and FADI pain subscores suggest 6 weeks of SFE training with the FootCore MAX can meaningfully reduce foot pain; the FAOS decreased by 10.5 points or 29%, which is greater than the minimal important change of 8.7 points [35]. Further, the clinically significant increase of 9 points in FAAM scores from pre‐training to post‐training highlights improved overall foot function [36]. As expected, we observed an increase in self‐reported pain from the beginning to the end of the pre‐training laboratory visit, as the participants were barefoot on a hard floor for an extended period, performing balancing and walking tasks. We did not observe an increase in pain scores due to laboratory testing in the post‐training visit, suggesting that training with the FootCore MAX could mitigate acute movement‐evoked pain. Reductions in movement‐evoked pain from the walking trials may represent improved tolerance to loading, as previously observed in exercise‐based rehabilitation in knee osteoarthritis patients [37]. This suggests that the FootCore MAX device may allow people with foot pain freedom to increase their daily physical activity with less movement‐evoked pain, as foot pain can limit physical activity [10]. Our results are consistent with previous literature showing that SFE decreases foot pain and disability in participants with pes planus [9]. It is important to note that our study is the first to use an SFE device, which promotes standardization of protocol for future IFM strengthening research. As previously mentioned, there is a need for standardized protocols, as there are varied interventions in SFE programs [1].

Surprisingly, we did not observe any foot posture improvements in AHI or ND, conflicting with the body of literature on SFE [1, 5, 6, 15, 18, 23] warranting further studies on this device. This could be due to the heterogeneity of our small sample; we included individuals with either pes planus or foot pain, while most studies indicate that SFE improves foot posture in populations with clinically diagnosed pes planus.

Furthermore, we did not observe any changes in static balance, which refutes our hypothesis of improved single‐leg balance. Improved balance is often reported after SFE training programs [1, 14, 18, 21]; Lynn et al. [18] found improvements in both static single‐leg balance and dynamic balance via the Y‐Balance test, while other studies [1, 14, 21] indicated only improved dynamic balance via the Y‐Balance test or Star Excursion test. Seven of the 13 participants in our study could not maintain 30 seconds of single‐leg standing without touching the contralateral foot to the ground, so a dynamic balance assessment with shorter single‐leg stance phases may have been more appropriate for evaluating improvements after the 6‐week training period. Other possible explanations for the lack of significant changes observed in single‐leg balance measures include too short of an intervention duration and the participants ability to perform the exercise correctly—apart from the first session, participants were not coached on their form over the course of the 6‐week intervention period.

As hypothesized, preferred gait speed increased after training with the FootCore MAX, likely due to decreased foot pain and improved foot function. These results are consistent with other studies connecting decreased pain with increased gait velocities [11, 12]. To our knowledge, our study is the first to observe increased preferred gait speed through SFE training in individuals with foot pain. The increased gait speed in our study conflicts with Sulowska‐Daszyk et al. [23], who found a decrease in comfortable walking speed after long distance runners participated in a 6‐week SFE program, although participants in their study did not report foot pain [23]. Importantly, our young adult sample initially walked at a mean speed of 1.06 m/s, which is slower than that of normative, able‐bodied adults aged 70–85 who walked at 1.10 m/s [38]. The increase from 1.06 m/s pre‐training to 1.16 m/s post‐training indicates improved gait speed, as a higher comfortable gait speed typically indicates better health [39].

Our study is not without limitations. As mentioned, the heterogeneity of our participants, who presented with either pes planus or foot pain, is a limitation of the study. Another limitation was the lack of control over participants' physical activity outside of the study; one participant noted that their feet were bothering them from wearing high heeled shoes for an extended time the night before the final lab visit, which potentially affected the pain results. A potential limitation of training with the device is the lack of standardization and directed periodization in the suggested recommended SFE training protocol. Some participants reported completing their exercises while sitting, while others reported standing. Participants were encouraged to complete the 4 minutes per day, in their choice of position, whether that was seated or standing. In contrast to our methods, Unver et al. [9], progressed their SFE program, by increasing the difficulty of the SFE throughout the 6 weeks of training. Future studies of the FootCore MAX device could evaluate differences in progressive versus nonprogressive SFE training, as our study only advised participants to use the device 4 minutes per day (2 minutes per foot per day) with no progression or periodization. Previously, varied methods (i.e., marble pick‐ups, towel curls, traditional arch‐raises) have been used to train the IFM, which complicates comparisons [1, 18]. Future studies should compare the use of the FootCore MAX device to a control group focused on typical SFE training. Additionally, exercise program compliance is positively influenced by access to at‐home equipment [40], which points to using an SFE device instead of traditional protocols to promote increased adherence.

In conclusion, 6 weeks of SFE training with the FootCore MAX device decreased pain in participants with chronic foot pain. Furthermore, there was reduced movement‐evoked pain in the participants' feet after the intervention, leading us to believe that using the FootCore MAX has the potential to positively impact participants' daily lives through a lessened pain response during and after physical activity. Increased preferred walking speed may indicate improvement in overall function and health, which should not be overlooked. The benefits of strengthening the IFM, with an at‐home device such as the FootCore MAX, offer a noninvasive training option for patients with foot pain.

## Author Contributions

**Yale B. Hughes:** data curation, formal analysis, writing – original draft. **Ashlyn M. Jendro:** methodology, project administration, writing – reviewing and editing. **Avery G. Dilley:** investigation, data curation, writing – original draft. **Elizabeth A. Parke:** methodology, writing – reviewing and editing. **Abigail C. Schmitt:** conceptualization, funding acquisition, resources, investigation, supervision, writing – reviewing and editing.

## Ethics Statement

This study was approved by the University of Arkansas Institutional Review Board (protocol # 2108353831) and consent was obtained from each participant.

## Conflicts of Interest

The authors declare no conflicts of interest.

## Data Availability

The data that support the findings of this study are available from the corresponding author upon reasonable request.
